# Comprehensive Analysis of Secondary Metabolites of Four Medicinal Thyme Species Used in Folk Medicine and Their Antioxidant Activities In Vitro

**DOI:** 10.3390/molecules28062582

**Published:** 2023-03-12

**Authors:** Rui Yang, Yanmei Dong, Fei Gao, Jingyi Li, Zora Dajic Stevanovic, Hui Li, Lei Shi

**Affiliations:** 1 Key Laboratory of Plant Resources, Institute of Botany, Chinese Academy of Sciences, Beijing 100093, China; yangrui1993@ibcas.ac.cn (R.Y.); dongyanmei@ibcas.ac.cn (Y.D.); gaofei1025@ibcas.ac.cn (F.G.); jingyileesd@126.com (J.L.); 2 China National Botanical Garden, Beijing 100093, China; 3 University of Chinese Academy of Sciences, Beijing 100049, China; 4 Department of Agrobotany, University of Belgrade Faculty of Agriculture, Nemanjina 6, 11080 Zemun, Serbia; zoradajicstevanovic@gmail.com

**Keywords:** *Thymus*, extract, phenolic acids, flavonoids, antioxidant activity, metabolites

## Abstract

Thyme is a colloquial term for number of aromatic species belonging to the genus Thymus L., known for their expressed biological activities and therefore used worldwide for seasoning and in folk medicine. In the present paper, the content of the total polyphenols (TP), total flavonoids (TF), and antioxidant capacity were assessed in the extracts of four traditionally used thyme species. Moreover, a comprehensive metabolomic study of thyme bioactive compounds was performed, and the obtained data were processed using multivariate statistical tests. The results clearly demonstrated the positive correlation between the content of the TP, TF, and antioxidant activity, and TF was more significant than TP. The findings revealed that four selected thyme species contained 528 secondary metabolites, including 289 flavonoids and 146 phenolic acids. *Thymus marschallianus* had a higher concentration of active ingredients, which improve its antioxidant capacity. Differentially accumulated metabolites were formed by complex pathways such as flavonoid, flavone, flavonol, isoflavonoid, and anthocyanin biosynthesis. Correlation analysis showed that 59 metabolites (including 28 flavonoids, 18 phenolic acids, and 7 terpenoid compounds) were significantly correlated with obtained values of the antioxidant capacity. The results suggested that selected thyme species exhibit a great diversity in antioxidant-related components, whereas flavonoids may be responsible for the high antioxidant capacity of all studied thyme species. The present study greatly expands our understanding of the complex phytochemical profiles and related applications of selected medicinal plants.

## 1. Introduction

Thymus L. is among the most important genera of medicinal plants of the Lamiaceae family, comprising over 200 species native to Europe, Asia, and North Africa [1,2]. Plants of the genus *Thymus* are traditionally used in foods and folk medicine and have been researched since ancient times. Studies on thyme species’ phytochemistry have revealed that their secondary metabolites, such as flavonoids, phenolic acids, terpenoids, lignin, and coumarins, are significant sources for treating digestive, circulatory, nervous respiratory, and other disorders [3,4,5]. In addition, polyphenols and flavonoids have excellent antioxidant, antibacterial, and anticancer functions [6,7], and have been extensively used in the food industry for our daily diet [8]. Former studies have found that reactive oxygen species (ROS) are related to various diseases such as atherosclerosis, cardiovascular diseases, diabetes mellitus, cancer, and so on [9,10]. Additionally, epidemiological studies have confirmed that the incidence of oxidative-stress-related conditions is lowered by the consumption of fruits and vegetables rich in compounds possessing high antioxidant activity [11,12]; thus, we would like to investigate the potential of these four thyme species in the health industry. Considering their long period of use in human history, it is important to further explore the valuable components of medicinal thyme species, originating from and distributed across different regions.

In *European Pharmacopoeia* 8.0, *Thymus vulgaris* L [13]. and *Thymus serpyllum* [14] were quoted as being thymi herba and wild thyme, respectively, exhibiting strong health-related effects. In *Chinese Pharmacopoeia* and *Xinjiang Medicinal Flora*, *Thymus quinquecostatus* Celak [15], and *Thymus marschallianus* Willd [16] are listed as the sources of medicinal plants. Considering the growth form, two prominent growth types of the thymi herba raw material (*Thymus vulgaris*) were mainly targeted: the erect-type and the creeping-type. Erect-type thyme is taller and easier to harvest, and is widely cultivated in Europe and the United States for pharmaceutical, food, and cosmetic applications, according to the *US Pharmacopoeia* and *European Pharmacopoeia* [17]. Creeping-type thyme is known to enhance microbiological properties and known for preventing the soil erosion thanks to the extremely robust and developed roots [18]. Given the previous work of our research group on thyme species [19,20], we found a different evolutionary background relating to their natural geographical distribution. For this reason, the similarities and differences in the components of erect-type and creeping-type thyme species and the differences in their pharmacological functions have aroused our interest.

Metabolomics is used for the study of small molecular metabolites of samples under certain physiological conditions, which is important for an assessment of their pharmaceutical effects and related drug quality control [21,22]. Most of the previous studies on thyme species were focused on the analyses of their essential oils [3,23], whereas recent studies revealed the importance of other secondary metabolites for pharmacological activities [24]. Some researchers focused on the quantity and qualitative analyses of several already known main compounds [15,16], where the global metabolic profiles of the species were missing. Due to the high separation power of the liquid chromatography and the great sensitivity of mass spectrometry (LC-MS), mass spectrometry with ultra-high phase liquid chromatography (UPLC) has become an increasingly popular approach for the qualitative and quantitative evaluation of plant secondary metabolites [25]. A widely targeted metabolomic method combines the advantages of targeted and nontargeted metabolite detection technologies with a high sensitivity, high qualitative accuracy, high throughput, great repeatability, and availability of comprehensive databases [26].

In this work, the four medicinal species of the genus Thymus (*T. serpyllum*, *T. vulgaris*, *T. quinquecostatus,* and *T. marschallianus*) were examined for their total polyphenol and total flavonoid concentrations, as well as their antioxidant activities. Furthermore, the secondary metabolites of the samples were profiled using a widely targeted metabolomics method. The goal of our work was to enrich the existing knowledge on the chemical profiles of different thyme species, and to address their future uses related to antioxidant activity and biological effects.

## 2. Results and Discussion

### 2.1. Total Polyphenol and Flavonoid Content

The content of the total polyphenols (TP) and total flavonoid (TF) was determined from thyme ethanolic extracts. The TP contents were 49.80 ± 0.10, 64.4 ± 2.10, 64.78 ± 0.20, and 64.50 ± 0.31 mg gallic acid equivalent/g of plant dry weight (mg GAE/g DW) in *T. serpyllum* (TS), *T. vulgaris* (TV), *T. quinquecostatus* (TQ), *T. marschallianus* (TM), respectively (Table 1). Additionally, the content of TF in TS was the lowest and significantly different from that in TV, TQ, and TM. The TF contents were 182.0 ± 0.24, 217.4 ± 1.18, 225.3 ± 1.20, and 279.0 ± 0.56 mg rutin equivalent (RU)/g of plant dry weight (mg RU/g DW) in TS, TV, TQ, and TM, respectively, and there are significant differences between the four thyme ethanol extracts (Table 1). These values prove that the erect-stem species have better activity than that the creeping-type thyme from the same place of origin (Europe: erect-stems species TV > creeping-stems species TS; East: erect-stems species TM > creeping-stems species TQ). This result was consistent with a previous study on cauliflower [27]. 

### 2.2. Antioxidant Activity

In the study, the antioxidant activity of thyme ethanolic extracts was detected using three methods: 2,2-diphenyl-1-picrylhydrazyl radical scavenging ability (DPPH) free-radical clearance rate, ferric reducing antioxidant power (FRAP), and 2,2′-Azinobis-(3-ethylbenzthiazoline-6-sulphonate (ABTS) antioxidant power. The FRAP and ABTS antioxidant power of extracts were expressed using the trolox equivalent (TE) (Table 2). The DPPH free-radical scavenging process yielded values of 76.3 ± 0.87, 74.2 ± 0.22, 74.8 ± 0.32, and 80.7 ± 0.65 of free radical clearance rate in TS, TV, TQ, and TM, respectively (Table 2). Additionally, the best DPPH scavenging property was shown for TM (80.7 ± 0.65%). The results of the FRAP test resulted in values of 152.33 ± 1.63, 165.00 ± 1.74, 160.67 ± 1.32, and 187.67 ± 1.65 μmol TE/g DW in TS, TV, TQ, and TM, respectively (Table 2). The antioxidant activity of ethanolic extract from TM was the strongest in the FRAP test, which was consistent with the results of DPPH. However, the results of FRAP showed that the worst activity was from TS ethanolic extracts, while the results of DPPH were from TV ethanolic extracts. The results of the ABTS test resulted in the values of 1.41 ± 0.21, 1.50 ± 0.03, 1.62 ± 0.16, and 1.54 ± 0.02 mmol TE/g DW in TS, TV, TQ, and TM, respectively (Table 2). The best antioxidant activity detected via ABTS is from TM ethanolic extracts. These results shown that the four thyme extracts had high antioxidant activity. However, the same extracts had a different contribution to DPPH, FRAP, and ABTS activity. For DPPH, small molecules may have a better chance to access the radical with a subsequently higher TAC value [28]. The ethanolic extracts of four thyme species contain different compounds and the same compounds have different contents. Therefore, it was challenging to compare the antioxidant potency of the four surveyed thyme species upon the results obtained from different antioxidant capacity methods. We used a suitable alternative (antioxidant potency composite, APC) to comprehensively evaluate the antioxidant activity of thyme ethanolic extracts according to previous studies [29,30]. The results showed the APC index of DPPH, FRAP, and ABTS were 87.6%, 90.8%, 92.8%, and 98.4% in TS, TV, TQ, and TM, respectively, following same pattern for the content of TF. These results suggest that the antioxidant activity of thyme ethanolic extracts may be related to the content of flavonoids.

**Figure 1:**
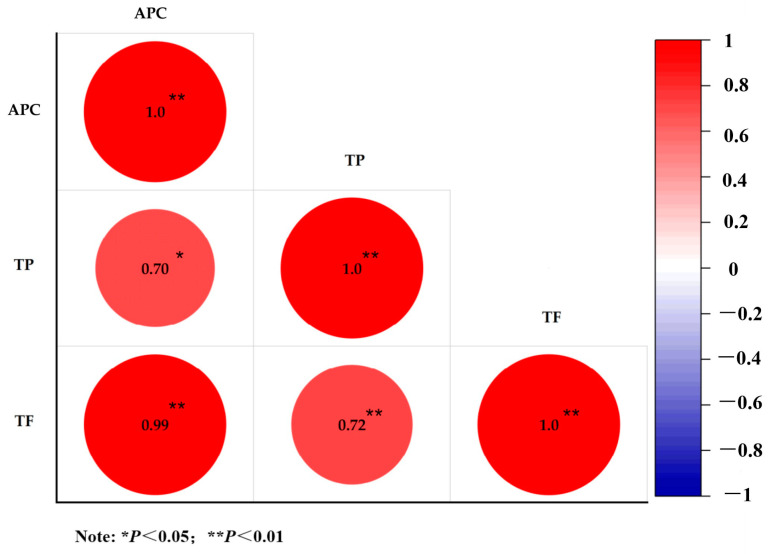
Pearson correlation plot of contents of TP and TF and APC. The content of total polyphenols (TP), total flavonoids (TF) and the antioxidant potency composite index (APC) of DPPH, FRAP, and ABTS were analyzed using Pearson correlation. Asterisk (*) indicates significant differences (* *p* < 0.05, ** *p* < 0.01).

Furthermore, the correlations between the content of TP, TF, and the APC index were analyzed. For antioxidant activity (APC), the results showed the Pearson correlation coefficient was 0.99 for TF, which was significant at the level of *p* ≤ 0.01. However, the Pearson correlation coefficient was 0.70 for TP, which was significant at the level of *p* ≤ 0.05 (Figure 1). The results illustrate that TF play a more pivotal role in the antioxidant activity of thyme ethanolic extracts. It was also shown that the TP content was highly related to the TF content (*p* ≤ 0.01). This result may be due to the biosynthesis relations of flavonoids and phenolic acids in plants, where many flavonoids occur in downstream biosynthesis pathways of phenolic acids [31,32].

### 2.3. Profiling of Secondary Metabolites

**Figure 2:**
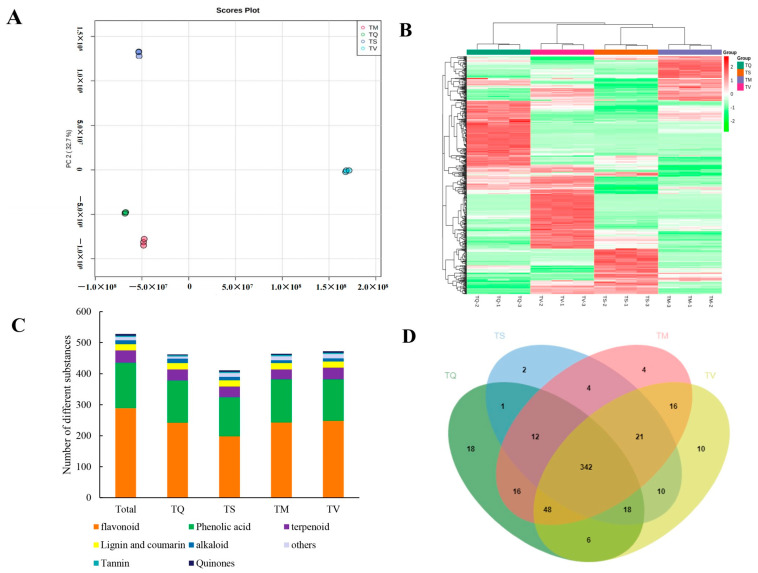
Overview of metabolites analysis detected in four thyme species. TQ: *T. quinquecostatus*, TS: *T. serpyllum*, TM: *T. marschallianus*, TV: *T. vulgaris*. (**A**) PCA analysis of the metabolites of TQ, TS, TM, and TV. (**B**) Cluster heatmap of metabolite content in different samples. (**C**) Distribution of substances in different thyme material. (**D**) Venn diagram of metabolite distribution in different thyme species.

Based on the results of the contents of TP and TF and related antioxidant activity, the range of secondary metabolites was analyzed. The multi-reaction monitoring peak graph exhibited all compounds that were detected in each sample (Figure S1), and we evaluated targeted substances by extracting ion chromatograms (XICs) and EPI for each Q1 (*m/z* ± 0.2 Da); accurate (*m/z*) mass spectra, and the XIC and EPI spectrum of acacetin-7-O-glucuronide are shown in Figures S2 and S3. The profiles of metabolites detected in the four selected thyme species are presented in Figure 2, Supplementary Table S1. The classification of secondary metabolites of different thyme species was analyzed using principal component analysis (PCA). The first PC axis and the secondary PC axis explained 80.6% of the overall variance (Figure 2A). Chinese thyme—TQ and TM—was clearly separated from TV according to PC1 (47.9%), and from TS according to PC2 (32.7%). In conclusion, the metabolic variation between TQ, TM, TV, and TS were obviously high, whereas TQ and TM showed presence of the similar metabolites (Figure 2A, Figure S4 and Table S2). Temperature, precipitation, and soil characteristics varied a lot between different habitats, affecting the types and quantity of secondary metabolites [32,33,34,35], in addition to the species-specific metabolomic profile. The high connection and differences between species were depicted from the heat map between samples (Figure 2B). Figure 2C showed that 528 secondary metabolites were identified by widely targeted metabolomics, referring to 289 flavonoids, 146 phenolic acids, 40 terpenoids, 20 lignin and coumarin compounds, 13 alkaloids, 6 quinones, and 10 other compounds. Detailed information on the metabolomic profiles is shown in Table S1. Moreover, 462, 411, 469, and 472 compounds were detected in TQ, TS, TM, and TV, respectively, and eight types were used to categorize all metabolites (Figure 2C). The main secondary metabolites in thyme are flavonoids and phenolic acids, which make up more than 80% of all identified compounds (Figure 2C). Furthermore, the TQ, TM, and TV exhibited more numerous and higher contents of some active substances compared with TS (Figure 2B,C). Concerning evaluated antioxidant capacity (Table 2), it could be suggested that the quantity and the content of metabolites strongly affect antioxidant activity. Only a few metabolites were exclusively found in a single studied species (in TS 2, in TM 4, in TV 10, and in TQ 18 species-related compounds), while 342 molecules were shared between four surveyed thyme species as shown in a Venn diagram, demonstrating a significant genus-dependent commonality in metabolic composition (Figure 2D). Most of the components was shared between the different thyme species, which is in line with an earlier study targeting metabolites in close species [36,37,38,39]. In addition, the four compounds specific to TM are Genistein-7-O-Glucoside, Salvianolic acid A, Petunidin-3-O-(6″-O-p-Coumaroyl)glucoside and Luteolin-7-O-glucuronide-(2→1)-(2″-Feruloyl)glucuronide. These four compounds, which have a strong antioxidant activity [40,41,42], may be the reason why the antioxidant activity of TM is stronger than that of other species.

### 2.4. Difference Analysis of Secondary Metabolites

**Figure 3:**
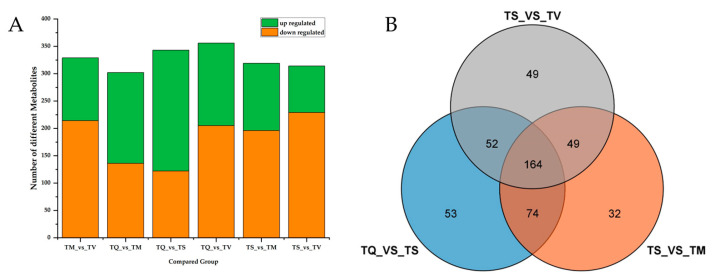
Differentially accumulated metabolites (DAMs) analysis. TQ: *T. quinquecostatus*, TS: *T. serpyllum*, TM: *T. marschallianus*, TV: *T. vulgaris*. (**A**) Bar graph of number of upregulated and downregulated DAMs through pairwise comparisons. (**B**) Venn graph for DAMs from the pairwise comparisons of TS and three other materials (TV, TQ, TM).

Differentially accumulated metabolites (DAMs) were analyzed with a fold change (FC) of ≥2 and OPLS-DA VIP value of ≥1. Figure 3A displays the number of chemicals that were up- or down-regulated after pairwise sample comparison. The group TQ_VS_TM had the fewest DAMs, with just 302 metabolites (166 upregulated and 136 downregulated). The most DAMs were found in the group TQ_VS_TV, accounting for 356 (151 upregulated and 205 downregulated). Moreover, TS compared with TQ, TM, or TV, had a higher number of the downregulated DAMs than the other group. This result was consistent with the number of metabolites identified in the four thyme ethanolic extracts, showing that the minimum quantity of secondary metabolites was identified in TS. A total of 164 DAMS was shared between the four analyzed thyme species (TQ, TM, TV and TS), and the unique DAMs of TQ_VS_TS, TS_VS_TV, and TS_VS_TM were 53, 49, and 32, respectively (Figure 3B).

**Figure 4:**
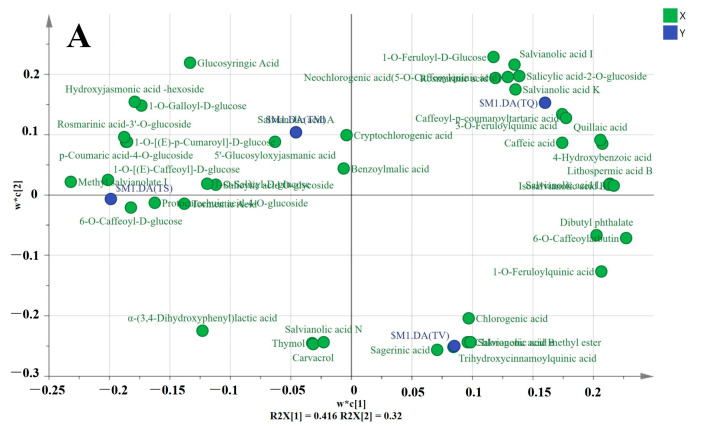

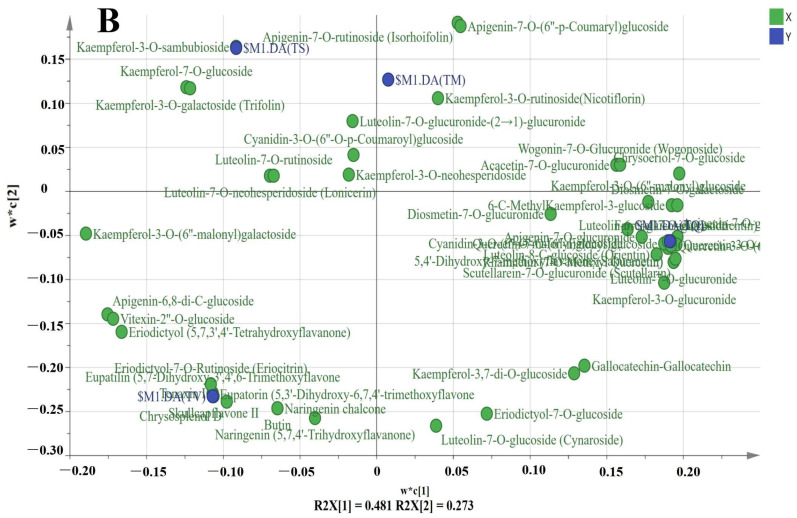
The compounds of metabolites that differentiate the four thyme species by VIP 1 (**A**) The landmark differential phenolic acid discriminating *T. quinquecostatus, T. serpyllum, T. marschallianus,* and *T. vulgaris* by VIP 1 (**B**) The landmark differential flavonoids discriminating *T. quinquecostatus, T. serpyllum, T. marschallianus,* and *T. vulgaris* by VIP 1.

From the PLS-DA loading plot (Figure 4), we identified the main compounds of metabolites that differentiate the four thyme species, a total of 92 significant metabolites were selected on the basis of VIP values (VIP > 0.85).

The phenolic acid discriminating TQ, TS, TM, and TV by VIP 1 were as follows (Figure 4A): benzoylmalic acid, cryptochlorogenic acid, salvianolic acid A, 5′-Glucosyloxyjasmanic acid, salicylic acid O-glycoside, 1-O-Salicyl-D-glucose, tormentic Acid, thymol, carvacrol, chlorogenic acid, salvianolic acid K, 6-O-Caffeoyl-D-glucose, neochlorogenic acid, sagerinic acid, caffeic acid, trihydroxycinnamoylquinic acid, and chlorogenic acid methyl ester.

The landmark differential flavonoids of four thyme species by VIP 1 were present as follows (Figure 4B): luteolin-7-O-glucuronide-(2→1)-glucuronide, luteolin-7-O-rutinoside, luteolin-7-O-neohesperidoside, luteolin-7-O-glucoside, apigenin-7-O-rutinoside, apigenin-7-O-(6″-p-Coumaryl)glucoside, kaempferol-3,7-di-O-glucoside, kaempferol-3-O-neohesperidoside, kaempferol-3-O-rutinoside, kaempferol-3-O-galactoside, kaempferol-3-O-sambubioside, kaempferol-7-O-glucoside, eupatorin, eupatilin, eriodictyol-7-O-glucoside, eriodictyol-7-O-rutinoside, maringenin, butin, naringenin chalcone, chrysosplenol D, tenaxin I, skullcap flavone II, diosmetin-7-O-glucuronide, and cyanidin-3-O-(6″-O-p-Coumaroyl)glucoside.

**Figure 5:**
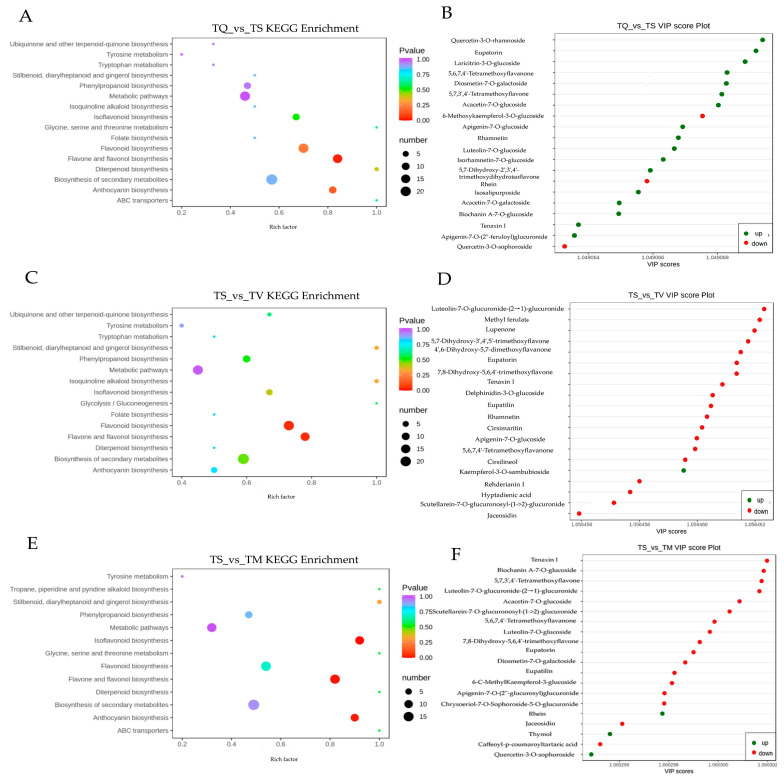
Enrichment analysis of differentially accumulated metabolites. TQ: *T. quinquecostatus*, TS: *T. serpyllum*, TM: *T. marschallianus*, TV: *T. vulgaris*. (**A**) Top 20 DAM-enriched KEGG pathways in TQ_VS_TS group. (**B**) Top 20 important variables in projections (VIPs) for TQ_VS_TS group. (**C**) Top 20 enriched KEGG pathways of DAMs in TS_VS_TV group. (**D**) Top 20 VIPs in TS_VS_TV group. (**E**) Top 20 DAM-enriched KEGG pathways in TS_VS_TM group. (**F**) Top 20 VIPs in TS_VS_TM group.

To study the metabolite pathway information in the selected thyme species, we performed KEGG enrichment analysis and variable importance analysis in projection of the DAMS of TS, comparing these parameters in TV, TQ, and TM. The top 20 DAM enrichment pathways are displayed in Figure 5A,C,E. The biosynthesis of flavonoids, flavones, flavonols, isoflavonoids, and anthocyanins was greatly enriched in the DAMs. Flavonoids, flavones, flavonols, isoflavonoids, and anthocyanins have strong antioxidant activity [43,44,45]. These findings might explain why TV, TQ, and TM had some higher levels of TP and TF, as well as better antioxidant activity. Figure 5B,D,F also showed that flavonoids are the major VIP constituents in three comparison groups. These results illustrate that flavonoids play a vital role in antioxidant activity. This coincides with the previous studies [45,46,47]. In addition, it was found that seven DAMS (Diosmetin-7-O-galactoside, 6-C-MethylKaempferol-3-glucoside, 5,2′-Dihydroxy-7,8-dimethoxyflavone glycosides, Luteolin-7-O-glucoside (Cynaroside), Salvianolic acid I, Quercetin-3-O-(4″-O-glucosyl) rhamnoside, Delphinidin-3-O-(6″-O-p-coumaroyl) glucoside) were common to all groups, suggesting that they may act as important antioxidant molecules (Figure S4 and Table S2).

### 2.5. Correlation Analysis of Secondary Metabolites and Antioxidant Capacity

**Figure 6:**
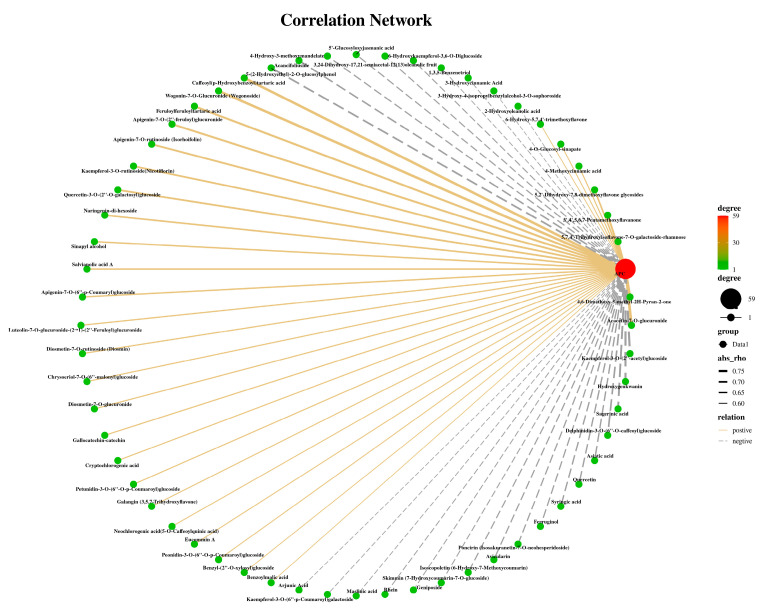
Correlation analysis of secondary metabolites and APC. Red circle indicates APC value, green circles indicate different metabolites, and the line shows the correlation; yellow line with positive correlation, grey line with negative correlation, and the greater correlation coefficient shown with a thicker line (*p* < 0.05).

The relationship between antioxidant capacity and 528 secondary metabolites was examined to study an impact of the secondary metabolites on values of antioxidant capacity and the results are shown in Figure 6. According to the performed correlation analysis, 59 metabolites were significantly correlated (*p* < 0.05) with APC. Among them, 31 metabolites, including 14 flavonoids, 10 phenolic acids, 2 anthocyanins, 1 flavanol, and 1 isoflavone, had a statistically positive correlation with APC (Figure 6 and Table S3). In addition, flavonoids were the most abundant substances in the secondary metabolites identified in the four thyme ethanolic extracts (Figure 2C). In the correlation analysis, Acacetin-7-O-glucuronide, 5,7,4′-Trihydroxyisoflavone-7-O-galactoside-rhamnose, 3′,4′,5,6,7-Pentamethoxyflavanone, Wogonin-7-O-Glucuronide, and Apigenin-7-O-(2″-feruloyl) glucuronide showed strongly positive correlations at a level of *p* ≤ 0.01 with APC. Previous studies have also shown that flavonoids have a strong antioxidant activity [43,44,45,46,47]. For example, Huang et al. found Wogon-in-7-O-Glucuronide alleviates colitis by improving the intestinal epithelial barrier function via the MLCK/pMLC2 pathway [48]. These results indicate that the strong antioxidant activity of thyme ethanolic extract may due to the flavonoids. Moreover, our results indicate that some terpenoids and other components of thyme, in addition to flavonoids and phenolic acids, are also significant antioxidants (Figure 6, Table S3).

From the results of the correlation analysis, the acacetin, apigenin, wogonin, and luteolin were the main active flavonoid aglycones, which are derived from the shikimic acid pathway and phenylpropanoid metabolism [49]. The structural characteristics for the antioxidant activity of typical flavonoids were discussed. The first is the catechol structure of the B ring [50], followed by the conjugated structure of the 2,3-double bond and the 4-oxygen functional group [51]. The presence of the 3-OH group and the 5-OH group contributes to forming a stable flavonoid structure [52]. These functional groups can exert antioxidant activity through hydrogen bond binding and electron transfer. Moreover, they can exert the antioxidant activity through metal chelation, and can also enhance electron transfer through delocalized electrons. The number, location, and degree of hydroxyl groups determine the antioxidant activity. The excellent antioxidant activity of quercetin [53] and other substances is closely related to their molecular structure. In thyme, the identification of 528 secondary metabolites provides a basis for the better understanding of antioxidant activity.

In addition, different antioxidant indexes of thyme were evaluated, and the principles of different antioxidant activity detection methods were different. The DPPH, ABTS, and FRAP used in this study were evaluated through the electron transfer method, and the antioxidant activity was evaluated through the detection of the value of the weakened color under the absorbance. In the detection of the total flavonoids, the Folin–Ciocalteu method has better affinity for flavonoids and flavonols, and the binding of flavanones and isoflavone is not stable, which deserves further attention in subsequent studies [54].

## 3. Materials and Methods

### 3.1. Plant Materials

**Figure 7:**
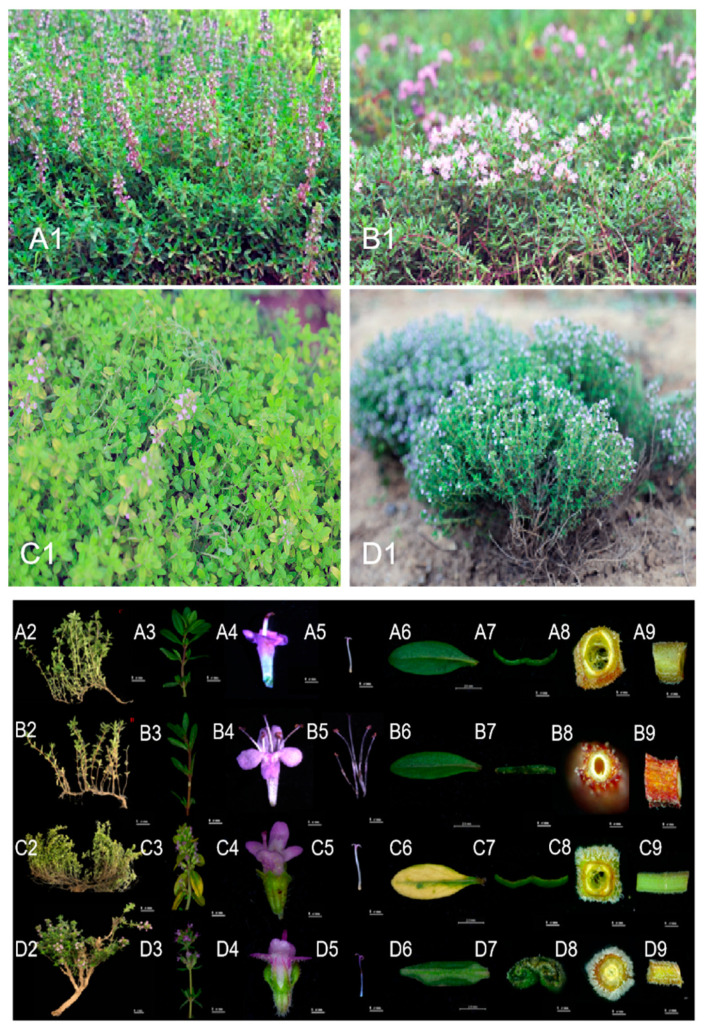
The material tested in the present study, and the morphological comparison between *T.marschallianus* (**A**), *T.quinquecostatus* (**B**)*, T.serpyllum* (**C**), *and T. vulgaris* (**D**). *Note:* Plant morphology (1); plant species traits and plant habitus (2); phyllotaxy (3); flower (4); stamens and pistils (5); blade (6); blade cross section (7); cross section of stem (8); lateral view of stem (9).

The four thyme species were obtained from the aromatic nursery plant germplasm of the China National Botanical Garden, situated at 39◦48′ N 116◦28′ E, located at the Institute of Botany of the Chinese Academy of Science, Beijing, China. Detailed information on the plant material is provided in Figure 7, more detailed morphological information of the four species is listed in Table S4.

### 3.2. Sample Preparation

The whole aboveground parts of the plants in the blooming period were harvested and put in liquid nitrogen immediately, then placed in a freeze-drying machine (Scienntz-100F) for vacuum freeze-drying for 72 h, and then ground to a powder using a grinder (MM 400, Retsch) for 30 Hz and 1.5 min. A total of 100 mg powder of each specie was weighed and dissolved in 1.2 mL of 70% methanol extract, vortexed 6 times in total for the extraction process, lasting 30 s each time with an interval of 30 min. The samples were placed in a refrigerator overnight at 4 °C, and then centrifuged at 12,000 rpm for 10 min, while the supernatants were sucked out and collected. The samples were filtered using a 0.22 μm membrane and kept in brown vials for further metabolite and antioxidant activity analysis.

### 3.3. Determination of Total Polyphenols and Total Flavonoids

The total polyphenols content was evaluated with the Folin–Ciocalteu method [54], with some modifications. In terms of mg gallic acid equivalents (GAE) per gram of dry weight, the data were presented using a standard curve using the equation *y* = 1.0407*x* + 0.0234, *R*^2^ = 0.999). The total flavonoid content was assessed with some modification of the assessment used in reference [55]. Absorbance was measured at 430 nm. The results were expressed as mg rutin equivalents (RU)/g of DW, calibration curve (*y* = 1.6277*x* − 0.0049, *R*^2^ = 0.999).

### 3.4. Antioxidant Activities

The test for DPPH radical scavenging activity was modified from the relevant literature [56]. Absorbance was measured at 517 nm. DPPH values were expressed as DPPH free radical clearance rate (%) (*y* = 3.0942*x* − 0.4082, *R*^2^ = 0.995). ABTS activity was assayed according to related references [56] with modifications. Absorbance was measured at 734 nm. ABTS activity was measured in mmol trolox equivalents (TE) per gram of dry weight (*y* = 2.59*x* + 0.012, *R*^2^ = 0.9997). FRAP activity was assayed according to related references [57] with modifications. Absorbance was measured at 593 nm. FRAP activity was expressed as μmol TE per gram of dry weight (*y* = 4.23*x* + 0.026, *R*^2^ = 0.9996). Each sample was assayed in triplicate. Based on three antioxidant assays results, API [29,30] was calculated according to the following equation: API = [(sample score/best score) × 100]%. The average values of all three tests were identified as APC.

### 3.5. UPLC-ESI-Q TRAP-MS/MS

The metabolite data were obtained using the UPLC-ESI-MS/MS system (UPLC, SHIMADZU Nexera X2; MS, Applied Biosystems 4500 Q TRAP; Agilent SB-C18 column (1.8 μm, 2.1 mm × 100 mm). Using the gradient elute technique, the mobile phase was composed of clean water with 0.1% formic acid (A) and acetonitrile with 0.1% formic acid (B). The gradient program was as follows: 0–9 min, B (5–95%) increased linearly; 9–10 min, B (95%) kept for 1 min; 10–11.10 min, B (95–5%) decreased linearly; 11.10–14 min, then B (5%) kept for 2.9 min. The flow rate was 0.35 mL/min with column oven at 40 ℃. The injection volume was 4 µL. A triple quadrupole linear ion trap mass spectrometer was used to acquire linear ion trap and triple quadrupole scans, and an ESI Turbo Ion-Spray interface was installed and was operational in both positive and negative ion mode. The metabolites data were analyzed with Analyst 1.6.3. The ESI parameters were set as follows: turbo spray (ion-source); source temperature (550 °C); ion spray voltage IS positive ion mode was set at 5500 V and ion spray voltage IS negative ion mode was set at −4500 V; ion source gas I set at 50 psi, ion source gas II set at 60 psi, ion source curtain gas set at 25 psi; collision-activated dissociation set at high; solutions of 10 and 100 μmol/L polypropylene glycol solutions were utilized to complete fine-tuning and mass calibration in the QQQ and LIT modes. According to the metabolites eluted during each phase, a particular set of MRM transitions were monitored.

### 3.6. Data Analysis

Triplicate analyses of the TP content, TF content, and antioxidant capacity were performed, and the results were represented as the mean with standard deviation (SD). Origin was used to depict correlation analysis (*p* < 0.05), heatmaps, principal component analysis (PCA) plots [58], and orthogonal partial least-squares discriminant analysis (OPLS-DA) (version 2021). DAMs were selected with a fold change of ≥2 and variable importance in projection value (VIP ≥ 1) from the OPLS-DA model. Kyoto Encyclopedia of Genes and Genomes (KEGG) pathway database (http://www.kegg.jp/kegg/pathway.html, accessed on 14 October 2022) was used to annotated metabolites, and differences were considered significant at *p* < 0.05.

## 4. Conclusions

The TP content, TF content, antioxidant capacity (APC of DPPH, FRAP and ABTS), and widely targeted metabolomics in four traditionally used thyme species were systematically researched for the first time. *T. quinquecostatus* and *T. marschallianus* had a higher content of TP and TF, and showed a higher antioxidant activity, followed by the *T. vulgaris* and *T. serpyllum* which exhibited a lower content of TP and TF, and a related antioxidant capacity. A total of 528 secondary metabolites, including 289 flavonoids and 146 phenolic acids, was profiled using widely targeted metabolomics. The antioxidant capacity was the strongest in *T. marschallianus*. KEGG enrichment analysis and variable importance in the projection of DAMS showed the biosynthesis of flavonoids, flavones, flavonols, isoflavonoids, and anthocyanins was greatly enriched in the DAMs. A total of 31 identified target compounds (including 14 flavonoids, 10 phenolic acids, 2 anthocyanins, 1 flavanol and 1 isoflavone) may be responsible for differences in the antioxidant activity of the studied species. Flavonoid content was rich in all thyme species, while the components varied a lot. The present study contributed to our understanding of the *Thymus* metabolomics, and addresses the meaningful bioactive potential of its species, indicating that numerous flavonoids highly contribute to the strong antioxidant activity and biological effects of the thyme.

## Figures and Tables

**Table 1:** The TP and TF content in ethanolic extracts of surveyed thyme species.

Species	TP (mg GAE/g DW)	TF (mg RU/g DW)
*T. serpyllum*	49.80 ± 0.10 b	182.0 ± 0.24 d
*T. vulgaris*	64.4 ± 2.10 a	217.4 ± 1.18 c
*T. quinquecostatus*	64.78 ± 0.20 a	225.3 ± 1.20 b
*T. marschallianus*	64.50 ± 0.31 a	279.0 ± 0.56 a
*p*-value	*p* < 0.01	*p* < 0.01

Note: TP: total polyphenols; TF: total flavonoid; GAE: gallic acid equivalent; QE: quercetin equivalent; DW: dry weight. Values are expressed as the mean ± SD. *p*-value: probability values obtained via one-way ANOVA; Different letters in the same line represent statistically different results (*p* ≤ 0.05) according to LSD.

**Table 2:** The antioxidant activity of ethanolic extracts of four surveyed thyme species.

Species	DPPH (% Inhibition)	FRAP (μmol TE/g DW)	ABTS (mmol TE/g DW)	APC (% Inhibition)
*T. serpyllum*	76.3 ± 0.87 b	152.33 ± 1.63 d	1.41 ± 0.21 d	87.6.0 ± 6.67 b
*T. vulgaris*	74.2 ± 0.22 dc	165.00 ± 1.74 b	1.50 ± 0.03 c	90.8 ± 2.54 b
*T. quinquecostatus*	74.8 ± 0.32 c	160.67 ± 1.32 c	1.62 ± 0.16 a	92.8 ± 7.20 b
*T. marschallianus*	80.7 ± 0.65 a	187.67 ± 1.65 a	1.54 ± 0.02 b	98.4 ± 2.83 a
*p* value	*p* < 0.01	*p* < 0.01	*p* < 0.01	*p* < 0.168

Note: DPPH: 2,2-diphenyl-1-picrylhydrazyl radical scavenging ability; FRAP: ferric reducing antioxidant power; ABTS: 2,2′-Azinobis-(3-ethylbenzthiazoline-6-sulphonate; APC: antioxidant potency composite index. Values are expressed as the mean ± SD. *p*-value: probability values obtained from one-way ANOVA; Different letters in the same line represent statistically different results (*p* ≤ 0.05) according to LSD.

## Data Availability

Not applicable.

## Supplementary Materials

The following supporting information can be downloaded at: https://www.mdpi.com/article/10.3390/molecules28062582/s1. Figure S1: The multi reaction monitoring peak graphs of TQ, TS, TV, and TM.; Figure S2: XIC of acacetin-7-O-glucuronide; Figure S3: EPI spectrum of acacetin-7-O-glucuronide; Figure S4: Venn graph for TOP 20 DAMs (FC > 2 and OPLS-DA VIP value < 1) from the pairwise comparisons between TV vs. TS, TQ vs. TS, and TM vs. TS; Table S1: Detailed metabolites data of four thyme species; Table S2: TOP 20 upregulated DAMs from the pairwise comparisons between TV_VS_TS, TQ_VS_TS, and TM_VS_TS; Table S3: Correlation analysis of secondary metabolites and APC.

## Sample Availability

Samples of the compounds are available from the authors.
