# Supplementary material for: Comprehensive Analysis of Secondary Metabolites of Four Medicinal Thyme Species Used in Folk Medicine and Their Antioxidant Activities In Vitro
Source: Molecules. 2023 Mar 12;28(6):2582. doi: 10.3390/molecules28062582 (PMC10052123; doi:10.3390/molecules28062582)
Supplement: Supplementary file 1 [file molecules-28-02582-s001.zip › Supplementary Figure.pdf]

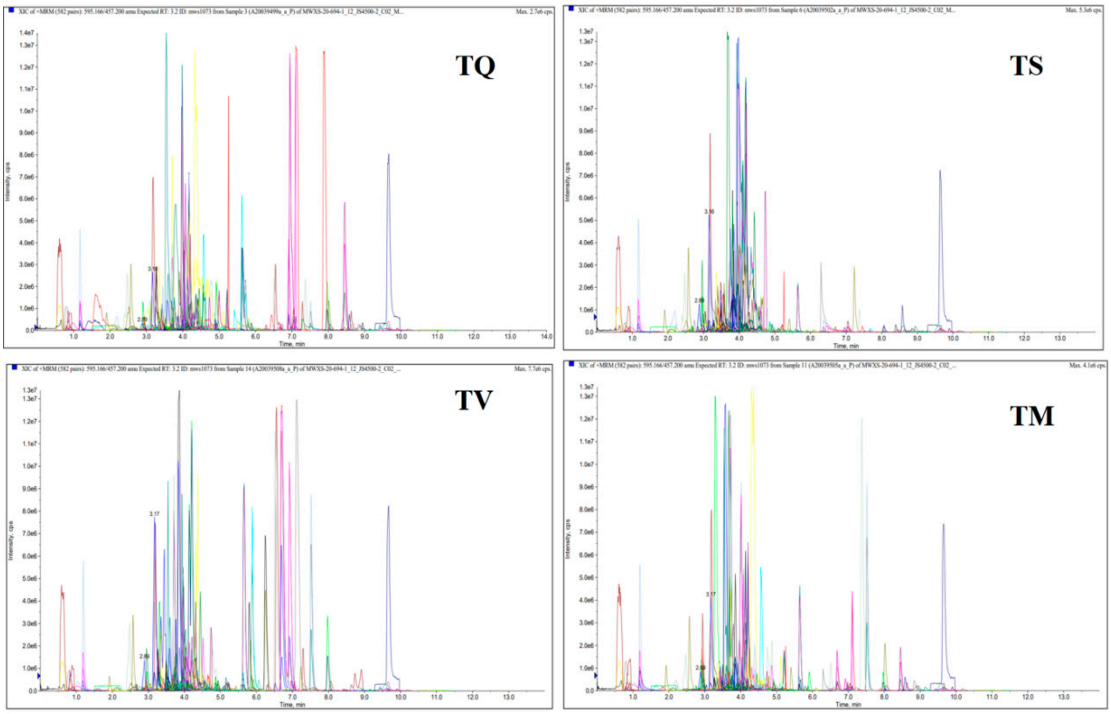

Figure S1. The multi reaction monitoring peak graphs of TQ, TS, TV, and TM.

TQ: *T. quinquecostatus*, TS: *T. serpyllum*, TM: *T. marschallianus*, TV: *T. vulgaris*.

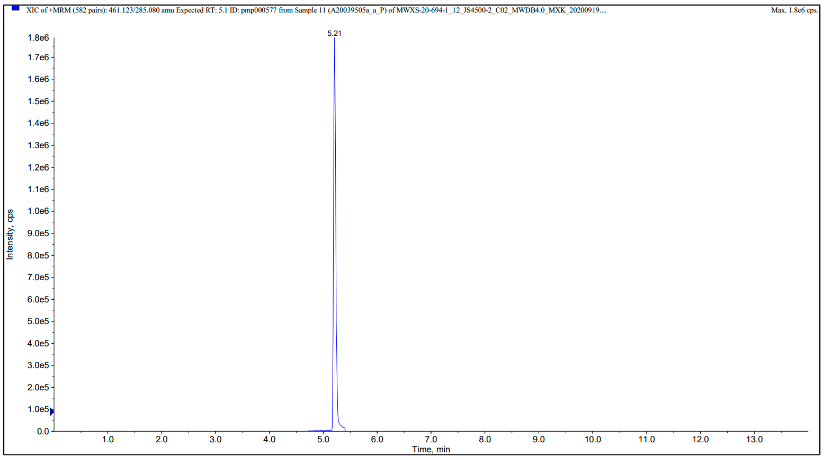

Figure S2. XIC of acacetin-7-O-glucuronide.

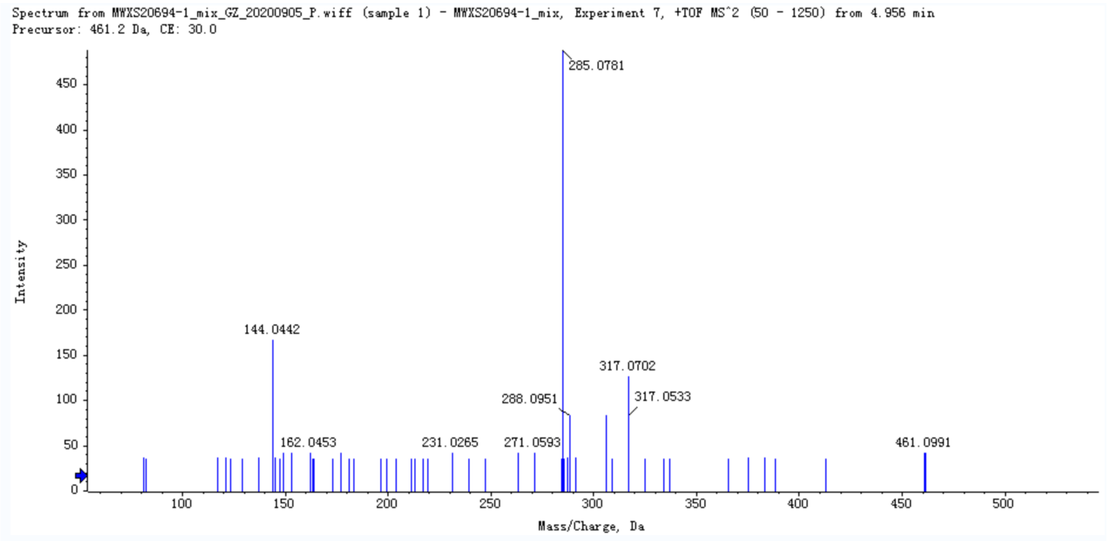

Figure S3. EPI spectrum of acacetin-7-O-glucuronide.

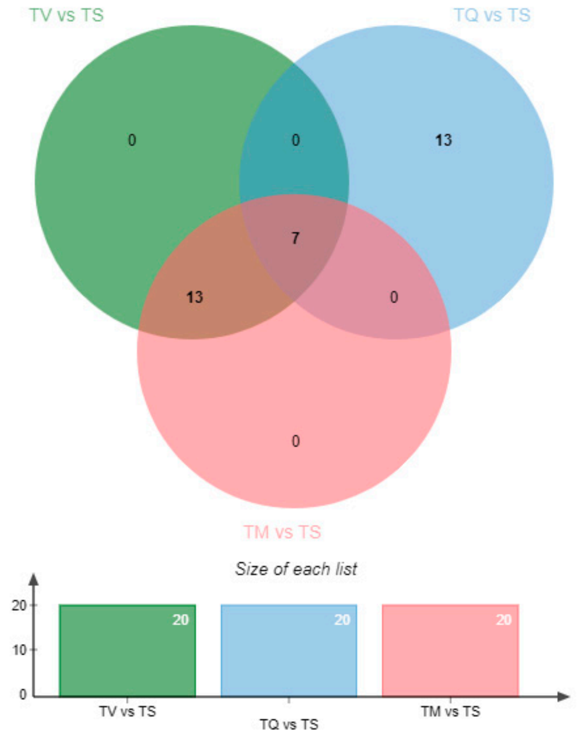

Figure S4. Venn graph for TOP 20 DAMs (FC>2 and OPLS-DA VIP value<1) from the pairwise comparisons between TV vs TS, TQ vs TS, and TM vs TS.
TQ: *T. quinquecostatus*, TS: *T. serpyllum*, TM: *T. marschallianus*, TV: *T. vulgaris*.
