# Supplementary material for: Comprehensive Analysis of Secondary Metabolites of Four Medicinal Thyme Species Used in Folk Medicine and Their Antioxidant Activities In Vitro
Source: Molecules. 2023 Mar 12;28(6):2582. doi: 10.3390/molecules28062582 (PMC10052123; doi:10.3390/molecules28062582)
Supplement: Supplementary file 1 [file molecules-28-02582-s001.zip › Table S4.pdf]

Table S4. The Morphological comparison among *T.marschallianus*, *T.quinquecostatus*, *T.serpyllum*, and *T. vulgaris*

Scientific Name	Plant morphology	Stamen	Blade Color	Blade Profile	Blade Curl	Stem Color	Stem Hair Density
T.marschallianus	Erect-type	Undeveloped	Green	Oval	Micro curl	Light green	Loose
T.quinquecostatus	Creeping-type	Developed	Green	Lanceolate	Flatten	Red	Loose
T.serpyllum	Creeping-type	Undeveloped	Yellow-green	Oval	Micro curl	Emerald green	Dense
T. vulgaris	Erect-type	Undeveloped	Green	Shield shape	Crimp	Yellow green	Dense
